# YouTube as a source of information on preventing the use of valproic acid in women during pregnancy

**DOI:** 10.1186/s12889-023-16036-5

**Published:** 2023-06-23

**Authors:** Boyang Qu, Binbin Kang, Xingyang Chen, Yanrong Ao, Liping Wang, Weiwei Cui

**Affiliations:** 1 Department of Neurology, Union Hospital, Jilin University, No. 126, Xiantai Street, Changchun, China; 2 Department of Nutrition and Food Hygiene, School of Public Health, Jilin University, No. 1163, Xinmin Street, Changchun, China; 3 Department of Neurology, the Affiliated Hospital to Changchun University of Chinese Medicine, Changchun, China

**Keywords:** YouTube™, Valproic acid, Valproate, Depakene, Depakote, Pregnancy

## Abstract

**Background:**

YouTube™ (http://www.youtube.com), as a very popular video site worldwide, is increasingly being used as a platform to disseminate health information. The purposes of this review were to assess the overall usefulness of the information on the prevention of valproic acid use in women during pregnancy on YouTube™ for patients.

**Methods:**

The YouTube™ website was systematically using 8 keywords. One hundred and fifty four videos meet the selection criteria. The researcher evaluated the video duration, days since upload, views and the likes. These videos are categorized as Education, News & Politics and People & Blogs. We designed a usefulness scoring scheme to assess videos quality and classified them as “slightly useful”, “useful” and “very useful”. The Kruskal-Wallis test was used to determine whether differences existed between total usefulness scores and categories and Pearson chi-square test for categorical variables.

**Results:**

The majority of videos were educational (62.8%). The "People & Blogs" and "News & Politics" videos scored significantly higher, but had no significant difference in days since upload, views, views/day or likes. More than half of the videos (91/154) were categorized as “useful”. The mean posted days (p = 0.045) was significantly different in the useful group compared with the slightly useful group. There were no correlation between usefulness and the number of days since upload, duration, views, views/day, or likes.

**Conclusion:**

YouTube™ is a promising source of information regarding the use of valproic acid during pregnancy. "News & Politics" videos are the most usefulness. Considering the presence of more slightly useful information, publishers need to improve more comprehensive video content that includes replacement medications, diagnoses and treatments. In the healthcare information space, consumers need to be directed to reliable video.

## Background

Valproic acid, a simple eight-carbon branched chain fatty acid, was initially used for seizure suppression. Thanks to its large spectrum of activity, its use has expanded to the treatment of other types of mental disorders, either alone or as an adjunct to other medications [1]. Valproic acid is currently used clinically for the treatment of epilepsy and bipolar disorder [2]. Although valproic acid proved to be a relatively safe drug, its side effects were quickly recognized. In the 1980s, valproic acid monotherapy during the first trimester of pregnancy was first associated with an increased risk of congenital spina bifida [3–6]. Subsequent studies have confirmed this increased risk and have also shown an increased risk of other major congenital malformations, including congenital malformations such as neural tube defects, atrial septal defects, and hypospadias in the fetus [7–11]. The study by Jäger-Roman E and Martínez-Frías ML also shows that the use of valproic acid during pregnancy causes major malformations in infants with a large number of typical minor craniofacial and finger malformations [11, 12]. In addition to teratogenic effects, the use of valproate during pregnancy has been associated with cognitive and behavioral impairment in infants [13–15].

The European Medicines Agency officially recommended in 2018 that valproate not be used by women during their reproductive years without a systematic method of contraception [16]. But the use of valproic acid during pregnancy, which causes congenital malformations in infants, is a raw topic for people with or without a medical background. Dissemination of high-quality information about valproic acid teratogenesis via social media may be a potential tool for educating physicians and general users interested in the topic. Specifically, for female patients of fertile age, access to more information could increase their awareness and thus make the prevention of valproic acid use during pregnancy easier to implement.

People are increasingly getting their health knowledge from the Internet, and people can easily and quickly search for the health information they want to know [17]. In fact, videos could be an effective tool for raising patient awareness, as it can provide information in a visual way, which could overcome some of the barriers to health literacy [18]. YouTube™, now recognized as one of the world’s most popular video sites, is increasingly being used as a platform for spreading health messages [17, 19]. However, the freedom of anyone to post videos has led to a mix of video content and a lack of professional review [20]. Therefore, it is crucial to assess the quality of the information delivered [21–25]. To our knowledge, no previous studies have investigated the use of YouTube™ as a source of video education for the prevention of valproic acid use in women during pregnancy. Therefore, this study aims to systematically evaluate the usefulness of YouTube™ videos in the prevention of valproic acid use in women during pregnancy in order to improve the professionalism of the video site.

## Methods

### Search strategy

Because this study involved only the use of public access data, it was exempt from Institutional Review Board’s approval of the study site. The method used was a systematic search via YouTube™ (http://www.youtube.com) on January 16, 2023, for videos containing information about valproic acid use during pregnancy. We searched for combinations of search terms related to valproic acid (i.e., “Valproic Acid”, “Valproate”, “Depakene” and “Depakote”) and pregnancy (i.e., “pregnancy” and “gestation”), according to Mesh (Medical Subject Headings, MeSH). The first 5 pages of each search result were filtered on the assumption that the user would not go beyond the first 5 pages of the search result [26]. We searched using YouTube’s default sorting option, “relevance”, which is considered to be probably the most commonly used option in YouTube’s sorting algorithm (relevance, upload date, number of views, rating). All the advertisements in the search results and at the beginning of the videos were neglected.

The videos that meet the inclusion criteria include: (1) in English; (2) available on January 16, 2023; and (3) about the use of valproic acid in women during pregnancy (videos related to tips for avoiding valproic acid, personal experiences with the drug, explanation of contraindications to valproic acid, etc). The exclusion criteria were:(1) videos that were not in English; (2) videos without sound; (3) advertisements; (4) videos with music, games, etc. that are not related to valproic acid use by women during pregnancy; and (5) videos that are partially or fully reproduced. Besides, multiple series or parts of the video were considered as one. It is worth noting that in order to avoid the statement that such professional content will be entertained to get the affirmation of the number of viewers or likes, the special ratings were made after the researchers and Professors Cui W. and Wang L. jointly agreed that the information was reliable.

All the included videos were saved in a document with Uniform Resource Locators (URLs), and the links and video titles for each video were organized into an Excel file as a backup. The researcher watched all the videos once before scoring each one for reliability and comparison.

### Video evaluation

We extracted information on the title, length of the video (in minutes), total number of views, days since upload, and likes of all videos that met the inclusion criteria. Calculations of views per day were performed. The included videos were divided into three categories [19]: Education; News & Politics; and People & Blogs. Specifically, “Education” includes medical courses or other academic videos; videos from government agencies and news reports about incidents or outbreaks of malformations caused by the use of valproic acid are classified as "News & Politics"; videos depicting personal experiences with fetal malformations caused by valproic acid use during pregnancy or videos showing personal views on valproic acid malformations were divided as "Personal & Blog".

The researcher scored the videos by rating both video quality and specific content. The Global Quality Scale (GQS) [27, 28] was used to assess the overall quality of all selected videos. As shown in Table 1, the GQS was a five-point Likert scale based on the quality of information, the flow, and ease of use of the information presented online. Seven additional content-specific items were developed to assess whether the videos discussed risk factors, epidemiology, etiology, symptoms, diagnosis, treatment, and prevention of valproic acid use during pregnancy. Depending on the content of each item in the video, the video was given a score of 0 point (Not mentioned), 1 point (Briefly introduced) and 2 points (Introduced in detail).The sum of the GQS score and the special content score is the total usefulness score. According to the total score, the videos were categorized as slightly useful (1–6), useful(7–13), and very useful (14–19).

**Table 1:** Global Quality Scale Criteria Used to Score Videos Containing Information about Prevention of Valproic Acid Use during Pregnancy.

Score	Global Score Description
1	Poor quality, poor flow of the site, most information missing, not at all useful for patients
2	Generally poor quality and poor flow, some information listed but many important topics missing, of very limited use to patients
3	Moderate quality, suboptimal flow, some important information is adequately discussed but others poorly discussed, somewhat useful for patients
4	Good quality and generally good flow, most of the relevant information is listed, but some topics not covered, useful for patients
5	Excellent quality and excellent flow, very useful for patients

### Statistical analysis

SPSS 24.0 was used for data entry and analysis. Categorical variables were expressed as frequencies and percentiles. Non-normally distributed continuous variables were expressed as quartiles (25th percentile, 50th percentile, and 75th percentile). The Kolmogorov-Smirnov test was used to test whether the data conformed to a normal distribution. The Kruskal-Wallis test was used to determine whether differences existed between total usefulness scores and categories and Pearson chi-square test for categorical variables [29]. A two-tailed p-value < 0.05 was considered statistically significant.

## Results

**Fig. 1:**
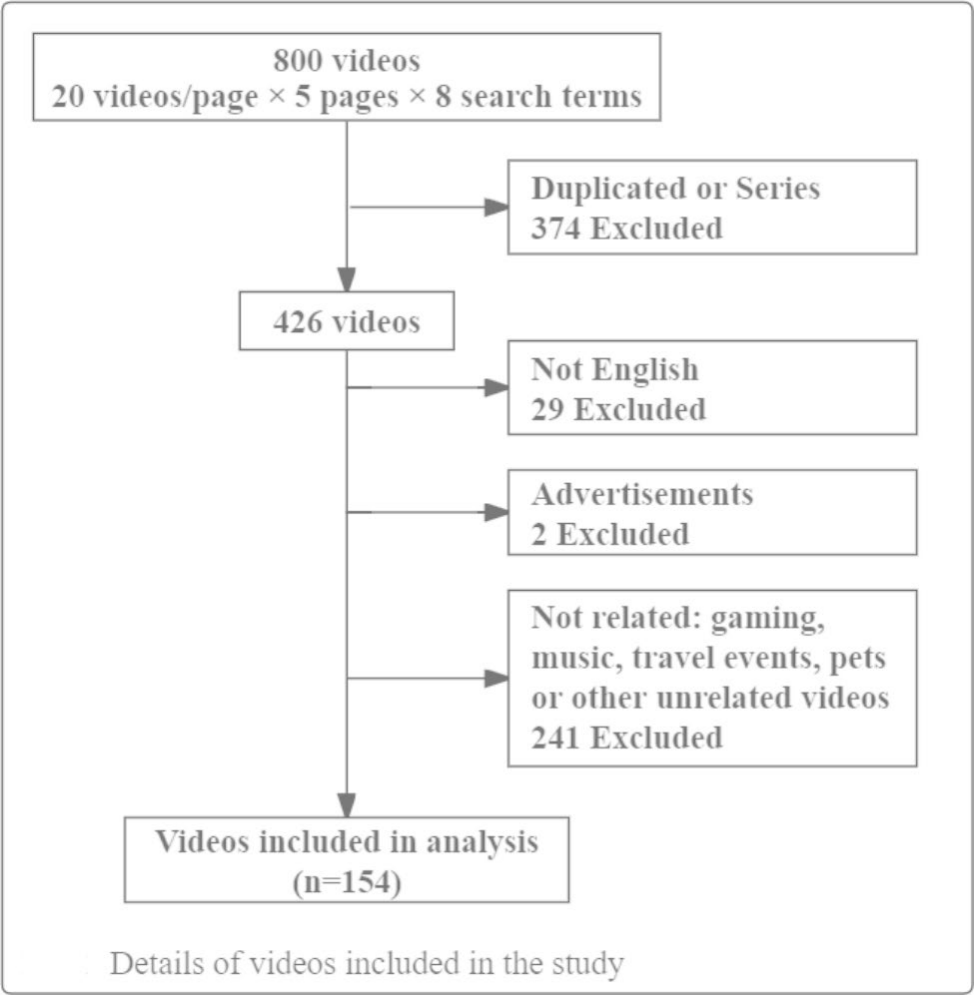
Details of videos included in the study.

As shown in Fig. 1, 100 videos were filtered based on each of the eight keyword combinations, and 154 unique videos met the inclusion criteria. The majority of videos were educational (n = 97, 62.75%), with "People & Blogs" (n = 37, 24.18%) being the second most common category. The total number of video views was 1545401 and the total duration was 2126 minutes. An overview of the included videos is shown in Table 2. The median duration of the videos was 6.0 min. Mean GQS score and content score of the videos was 2.8 ± 0.8, 4.6 ± 2.2 respectively. Mean total score of the videos included in the study was 7.4 ± 2.9.

**Table 2:** Summary of the YouTube™ video on the use of valproic acid during pregnancy.

Characteristics	Total (n = 154)
Category n (%)	
Education	97(62.75%)
People & Blogs	37(24.18%)
News & Politics	20(13.07%)
Posted days	1341.5(488.25,1958.25)
Duration	6.0(2.0,15.0)
Views	1098.5(196.75,4133.5)
Views/day	1.0(0.3,4.6)
Likes	8.5(1.0,46.5)
GQS score	3.0(2.0,3.0)
Content score	4.0(3.0,6.0)
Total score	7.0(5.0,9.0)

The videos are classified by category, and other features are detailed as shown in Table 3. There was no significant difference between category and posted days, views, views/day or likes (P = 0.228, P = 0.892, P = 0.448, P = 0.247). Video duration was correlated with category (p = 0.003).The duration of “Education” videos may be longer compared to "People & Blogs" and "News & Politics" videos (p = 0.006, p = 0.008). In addition, educational videos accounted for more than half of the total duration and total views. Of the content scores, 150 (97.4%) videos discussed risk factors, 58 (37.7%) videos mentioned epidemiology, 101 (65.6%) videos explained symptoms, 140 (90.9%) videos presented prevention, only 9 (5.8%) videos mentioned etiology, 7 (4.5%) videos analyzed diagnosis, and 13 (8.4%) videos provided information regarding treatment options. (both briefly introduced and introduced in detail). The Kruskal-Wallis test showed that content scores and total scores were correlated with category (P = 0.035, P = 0.048). The content score and total score of "News & Politics" videos may be higher compared to “Education” videos (P = 0.009, P = 0.012). There was no significant correlation between GQS scores and video category (p = 0.517).

**Table 3:** Detailed characteristics of videos based on category (median [quartile range]).

	Education (n = 97)	People & Blogs (n = 37)	News & Politics (n = 20)	H	P
Posted days	1343 (540,1960.5)	1097.0 (353.0,1751.5)	1683.0 (1128.3,2828)	2.959	0.228
Duration (minutes)	9.0 (3.0,27.5)	4.0 (1.5,7.5)	4.0 (2.0,6.75)	11.941	0.003^*^
Views	925.0 (173.5,5218.5)	1373.0 (413.0,3557.0)	1273.5 (215.5,3687.75)	0.229	0.892
Views/day	0.8 (0.2,5.1)	2.2 (0.4,5.4)	1.0 (0.2,2.7)	1.606	0.448
Likes	4.0 (1.0,30.5)	13.0 (3.0,57.0)	10.0 (2.3,25.0)	2.793	0.247
Total duration (minutes [%])	1787 (84.05%)	248 (11.67%)	91 (4.28%)	-	-
Total views (n [%])	1344441 (87.00%)	96765 (6.26%)	104195 (6.74%)	-	-
GQS score	3.0 (2.0,3.0)	3.0 (2.0,3.0)	3.0 (3.0,3.0)	1.32	0.517
Content score	4.0 (3.0,6.0)	4.0 (3.0,6.0)	5.5 (5.0,6.0)	6.688	0.035^*^
Total score	7.0 (5.0,9.0)	7.0 (5.0,9.5)	8.5 (8.0,9.8)	6.079	0.048^*^

*statistically significant

Videos were further categorized into “slightly useful”, “useful” and “very useful” based on their total usefulness score. Video demographics according to usefulness are shown in Table 4. More than half of the videos were useful (n = 91, 59.1%), while 53 of the videos (34.4%) were slightly useful, and only 10 of the videos (6.5%) were considered very useful. There was no significant difference between the groups regarding the number of “posted days”, “duration”, “views”, "views/day" and “likes”. The number of posting days was significantly higher in the useful group than in the slightly useful group (P = 0.045). Chi-square test showed no correlation between the usefulness category and video category (P = 0.340).

**Table 4:** Detailed characteristics of videos based on usefulness (median [quartile range]).

	Slightly useful (n = 53)	Useful (n = 91)	Very useful (n = 10)	H	P
Posted days	1097.0(451.0,1670.0)	1376.0(540.0,2252.0)	1810.5(512.3,2763.0)	4.726	0.094
Duration (minutes)	6.0(3.0,12.0)	6.0(2.0,17.0)	9.0(4.5,15.0)	1.999	0.368
Views	980.0(176.0,5702.0)	1227.0(246.0,3559.0)	1013.0(395.0,7766.0)	0.413	0.813
Views/day	1.5(.0.2,5.5)	0.9(0.3,3.9)	1.6(0.3,5.1)	0.571	0.752
Likes	6.0(1.0,49.0)	9.0(1.0,34.0)	15.5(2.0,23.5)	0.240	0.887
Total duration (minutes [%])	699(32.22%)	1427(63.73%)	113(5.05%)	-	-
Total views (n [%])	567762(35.32%)	977639(60.81%)	62166(3.87%)	-	-
Category				-	-
Education	37	54	6		
People & Blogs	13	21	3		
News & Politics	3	16	1		

## Discussion

This study is the first to explore the use of valproic acid in women during pregnancy on YouTube™. A total of 154 videos with a total of 35.4 hours and over 1.5 million total views were included in this study, and a detailed analysis of YouTube™ videos as a source of medical information was conducted. Total viewership was low compared to previous studies evaluating video content on other topics [28, 30]. Of the 154 videos, 91(59.1%) were useful, indicating that YouTube™ is an important tool for broadcasting useful information. The percentage of useful videos was higher compared to previous studies evaluating video content on other topics [31–34]. However, only 10 (6.5%) of the total videos are very useful, which means that although most of the videos are useful, the useful videos were not comprehensive. The results showed no significant differences in GQS scores between the categories. This is probably due to the better quality of most videos, but there was less content addressing the use of valproic acid during pregnancy and it was consistent between the groups.

In general, the videos included are mixed in terms of the detail they contain. Not surprisingly, the risk factors, epidemiology, symptoms, and prevention of valproic acid use during pregnancy have been discussed most frequently. Of these, risk factors (97.4%) and prevention (90.9%) were mentioned in almost every video. The etiology, diagnosis, and treatment have been less discussed. After watching all of the videos, the researcher concluded that some of the videos were simply summarized as "valproic acid is teratogenic" and "valproic acid should be avoided during pregnancy", with no details on alternative medications, diagnostic and treatment methods.

Of the 154 videos we studied, 37 (24.18%) were categorized as "People & Blogs", and most of these videos were about personal experiences or opinions about congenital malformations or mental disorders caused by valproic acid use during pregnancy. This suggests that individuals are using the Internet not only to seek health information but also to disseminate health and medical information. Some of the videos of food poisoning in the study by Li M [28] were classified as entertainment. In contrast, no videos were classified as entertainment in all videos of this study, which may indicate that valproic acid is used by a smaller population and is less prevalent. 97 (62.75%) were “Education” videos, most of which were medical courses and academic videos, and the length of the videos was generally very long. However, some of the educational videos pass over the fact that pregnant women should avoid valproic acid, which can easily be overlooked. Both "People & Blogs" and "News & Politics" scored higher than the “Education” category. Based on the content of the videos, researchers explained that some of the "People & Blogs" and "News & Politics" videos explain symptoms, causes, treatment and prevention based on their own experiences or social events. The "People & Bloggers" and "News & Politics" videos received more views than the “Education” videos, probably because most of these videos were about patients’ personal experiences or news reports. Such narratives are credible, more relevant, more serious, and easier to remember. There was no significant difference in the number of days posted, views, or likes between categories, probably because our search results were limited to the first five pages of videos based on “relevance” order.

After categorizing the videos as slightly useful, useful, and very useful, the results show that there is no difference in “likes” and “duration” between the different levels of usefulness, so “likes” and “duration” cannot be used to help determine the usefulness of a video. Moreover, we found that the “slightly useful” videos were uploaded more recently. This is certainly concerning and suggests that not only may users not be able to judge the quality of the information presented on YouTube™, but that some of the recent videos have gradually begun to ignore detailed explanations of valproic acid use during pregnancy. The reason for this may be that, as mentioned in the article by Di Vito L et al [35], the requirement to avoid the use of valproic acid in pregnant women has been introduced by the drug regulatory authorities in several countries in recent years, resulting in less and less use of valproic acid in women during pregnancy. However, the safe use of this drug can vary in different countries, and as you can see from this article and the content of the videos (https://youtu.be/JJygXxObLlI and https://youtu.be/MNv-BG-bgF0), there are still incidents of babies being deformed everywhere in the recently released videos. It is therefore worth noting that YouTube, as a widely used video distribution site worldwide, should still increase its detailed content and education on the avoidance of valproic acid during pregnancy.

Valproic acid is a very useful drug for epilepsy-like treatment. And with the study of new mechanisms of therapeutic and toxic metabolites of valproic acid in humans, the indications for valproic acid sodium have been expanding in recent years [36]. However, there is a significant teratogenic risk associated with the use of VPA during pregnancy, and adverse consequences are likely to occur if treatment regimens are not promptly adjusted according to physician recommendations. Therefore, health care providers should improve multimedia content by producing more comprehensive materials that are easy for internet users to understand and by sharing evidence-based video information. It should include the prevention of valproic acid use during pregnancy, appropriate replacement medications, the prevention of infant malformations in pregnant women who are already using valproic acid and early life care after causing malformations. Healthcare professionals and the general public should be aware of the potential role of YouTube as an educational tool [17].

There are several limitations to this study. First, using a customized usefulness scoring scheme to evaluate videos is subjective, as there is no validated tool to evaluate video data. Second, the results of the study may vary depending on the situation, and the search used keywords that were assumed to be what a layperson would choose for this study. This may not always be the case. Third, videos were sorted by relevance, which is the default setting of YouTube™. Search results may be influenced by ranking criteria. In addition, YouTube™ search results are dynamic, changing when new videos are uploaded or old videos are deleted. Thus, this cross-sectional study demonstrates the usefulness of the information on the prevention of valproic acid use in women during pregnancy at that time.

## Conclusions

In conclusion, there is a large amount of information on YouTube™ about the use of valproic acid by women during pregnancy. More than half of the YouTube™ videos regarding valproic acid use during pregnancy are useful and provide information on risk factors, symptoms, or prevention of valproic acid use during pregnancy. In general, "News & Politics" videos based on specific patients’ personal experiences and news reports are more useful in providing information on the use of valproic acid during pregnancy. Considering the presence of more slightly useful information, publishers need to improve video quality and provide more comprehensive video content, including replacement medications, diagnoses and treatments. And consumers need to be directed to reliable videos in the area of healthcare information.

## Data Availability

The datasets used and/or analysed during the current study are available from the corresponding author on reasonable request.

## Abbreviations

GQS: Global Quality Scale
URL: Uniform Resource Locators
